# Putative Role of CFSH in the Eyestalk-AG-Testicular Endocrine Axis of the Swimming Crab *Portunus trituberculatus*

**DOI:** 10.3390/ani13040690

**Published:** 2023-02-16

**Authors:** Mengen Wang, Rui Xu, Shisheng Tu, Qiaoling Yu, Xi Xie, Dongfa Zhu

**Affiliations:** Key Laboratory of Aquacultural Biotechnology Ministry of Education, School of Marine Sciences, Ningbo University, Ningbo 315822, China

**Keywords:** *Portunus trituberculatus*, CFSH, sex differentiation, endocrine axis, crustaceans

## Abstract

**Simple Summary:**

The crustacean female sex hormone (CFSH) plays a crucial role in the development of secondary sexual characteristics in female Decapoda crustaceans, but its physiological function in males is less reported. In the present study, the CFSH of *P. trituberculatus* (*PtCFSH*) was identified, cloned and expressed in the recombinant form. The physiological functions of *PtCFSH* on the AG and testis were investigated using the RNAi and recombinant protein injection in a series of in vivo and in vitro experiments. It was confirmed that *PtCFSH* can negatively regulate the spermatogenesis and testicular development via a CFSH-IAG-testis endocrine axis. PtCFSH may also act directly on the testis, dependent or independent of IAG. The signaling system of PtCFSH involves the participation of cAMP, cGMP, and NO, which resembles the MIH signaling pathway. In summary, our results demonstrated the mode of CFSH action in *P. trituberculatus*.

**Abstract:**

It has been shown in recent studies that the crustacean female sex hormone (CFSH) plays a crucial role in the development of secondary sexual characteristics in Decapoda crustaceans. However, research on the function of *CFSH* in the eyestalk-AG-testicular endocrine axis has been inadequate. We cloned and identified a homolog of *CFSH*, *PtCFSH*, in this study. RT-PCR showed that *PtCFSH* was mainly expressed in the eyestalk. A long-term injection of dsPtCFSH and recombinant PtCFSH (rPtCFSH) in vivo showed opposite effects on spermatogenesis-related gene expression and histological features in the testis of *P. trituberculatus*, and was accompanied by changes in AG morphological characteristics and *PtIAG* expression. In addition, the phosphorylated-MAPK levels and the expression of several IIS pathway genes in the testis was changed accordingly in two treatments, suggesting that PtCFSH may regulate the testicular development via IAG. The hypothesis was further validated by a mixed injection of both dsPtCFSH and dsPtIAG in vivo. The following in vitro studies confirmed the negatively effects of PtCFSH on AG, and revealed that the PtCFSH can also act directly on the testis. Treatment with rPtCFSH reduced the cAMP and cGMP levels as well as the nitric oxide synthetase activity. These findings provide vital clues to the mechanisms of CFSH action in both the eyestalk-AG-testis endocrinal axis and its direct effects on the testis.

## 1. Introduction

The traditional view is that sexual differentiation in crustaceans is a male-dominated process. For example, in crustaceans, a male-specific androgenic gland (AG) determines male sexual differentiation and the maintenance of secondary sexual characteristics; after the removal of the AG, crustaceans undergo feminization [1,2], and vice versa [3]. An essential hormone was later identified as an insulin-like androgenic gland hormone (IAG) in AG. Moreover, the Sagi group have achieved functional sexual reversal by chronically interfering with *MrIAG* expression in young *Macrobrachium rosenbergii* [4]. This also induced the cessation of spermatogenesis in the spermathecae and the incomplete development of secondary sexual characteristics. The function of silencing *IAG* in *Cherax quadricarinatu* and *Eriocheir sinensis* to affect the male phenotype was further validated [5,6]. Thus, IAG is referred to as the sexual “IAG-switch”, regulating the development of the male decapods [7].

Khalaila previously suggested that the eyestalk-AG-testicular endocrine axis occurs in decapod crustaceans [8]. Additionally, it has been suggested that the removal of the eyestalk leads to AG hypertrophy, accompanied by an increase in IAG mRNA in males [9,10,11], stimulates spermatogenesis and increases testicular weight [8,12]. Further studies confirmed that AG extracts could affect the phosphorylation of testicular polypeptides in vitro [8]. This demonstrates the direct effect of the eyestalk on the AG and testis, and the direct effect of the AG on the testis. The X-organ sinus gland complex (XO–SG) is located in the eyestalk of crustaceans. It is the primary site of neurohormone synthesis and secretion, which is released into the hemolymph to perform crustacean endocrine physiological functions.

Recently, several neuropeptides, such as the crustacean hyperglycemic hormone (CHH), the molt-inhibiting hormone (MIH), and the red pigment concentrating hormone (RPCH), have been found to be involved in crustacean development [13,14]. Among them, in freshwater prawn *Macrobrachium nipponense*, both the gonadal inhibitory hormone (GIH) and molt inhibitory hormone (MIH) have a repressive effect on the expression of *IAG* genes [15].Moreover, the crustacean female sex hormone (CFSH) was first identified and named in *Callinectes sapidus*, and is preferentially expressed in the eyestalk. It contains structures not found in other known neuropeptides, is considered unique to crustaceans, and has a regulatory role in secondary sexual characteristics [16]. *CFSH* has also been identified in several species, including *Sagmariasus verreauxi* [17], *C. quadricarinatus* [18], *Marsupenaeus japonicus* [19], *Scylla paramamosain* [20], *M. rosenbergii* [21] and *Lysmata vittate* [22]. Recently, it has been shown that the CFSH may act as an upstream regulator of IAG, and there is evidence for negative regulation. For example, the expression profile of *SpIAG* versus *SpCFSH* in *S. paramamosain* in the eyestalk during the AG developmental cycle showed an opposite pattern [20]. Additionally, recombinant SpCFSH was incubated in vitro with AG explants and was found to have a repressive effect on *SpIAG*. At the same time, a reciprocal inhibitory effect of *LvCFSH1* with *LvIAG* in *L. vittate* was revealed [22]. The same feedback regulatory loop was also found in *EsCFSH1* and *EsIAG* by in vivo injection into *E. sinensis* [23].

Although the negative regulatory effect of *CFSH* on *IAG* has been mostly described, the majority of reports on *CFSH* function are studied in females [16,22,23]. In male crustaceans, it was found that the disruption of the CFSH affects the male-associated phenotypes in *L. vittate* [22] and penis development in *E. sinensis* [23], suggesting that the function of the *CFSH* in males is also related to *IAG*. However, in *L. vittate*, the long-term RNA silencing of the *CFSH* fails to affect testicular maturation [22]; this raises questions about whether *CFSH* is involved in the “eyestalk-AG-testis” endocrine axis. Therefore, the function of the *CFSH* in male crustaceans needs to be studied in more species, and its mode of action and molecular mechanism need to be more clearly elucidated.

The swimming crab *Portunus trituberculatus* is an economically essential crab species on the southeast coast of China, but the endocrine regulatory mechanism of its testicular development remains largely unclear. In this study, the homolog of the *CFSH* in *P. trituberculatus* (*PtCFSH*) was cloned and characterized. The function of *PtCFSH* in male *P. trituberculatus* was studied using a series of in vivo and in vitro experiments, based on the double-strand RNA-mediated RNAi technology and recombinant-CFSH treatments. We confirmed that the PtCFSH is involved in the “eyestalk-AG-testis” endocrine axis, and can also act directly on the testis.

## 2. Materials and Methods

### 2.1. Experimental Animals

The experimental animal in this study is *Portunus trituberculatus*, which belongs to invertebrates and is a kind of crab. In China, ethical approval is not required for experiments on crabs. All of our crabs were purchased from the local aquatic market. Prior to dissection, we anesthetized the crabs on ice. All the experiments comply with the requirements of the governing regulation for the use of experimental animals in Zhejiang Province (Zhejiang provincial government order No. 263, released on 17 August 2009, effective from 1 October 2010) and the Animal Care and Use Committee of Ningbo University. Healthy wild adult swimming crabs with a body weight of 100 to 140 g were purchased from the local aquatic market in Xianxiang, Ningbo. Some individuals in the intermolt period were temporarily reared in a capacious 120 L tank bottom containing 30 cm of fine sand and shelter in our laboratory; the crabs were fed and their water was changed periodically until the 20-day (long-term) and 3-day (short-term) treatment of RNAi and the in vivo recombinant proteins injection was completed. Other crabs were collected for gene cloning and in vitro treatment, respectively. All samples were stored in RNA preservation fluid (Cwbiotech, Taizhou, China) at −80 °C until RNA extraction.

In addition, the testis and AG-related tissues were sampled from the crab and cut into 3–5 mm pieces and fixed in 4% Paraformaldehyde Fix Solution for 24 h at 4 °C. Then, the tissues were dehydrated with serial ethanol, cleared with serial xylene, and finally embedded in paraffin. Tissues were cut into 5–7 μm sections for hematoxylin and eosin (H&E) staining. Next, 30 to 50 mg of testis tissue was placed in a 1.5 mL EP tube containing 300 microliters of PBS and (Protease and phosphatase inhibitor cocktail for general use, 50×) (Beyotime, Shanghai, China), after which the homogenate was centrifuged at 8000 *g* for 10 min at 4 °C. The supernatant was sucked and stored at −80 °C for western blotting.

### 2.2. Extraction of Total RNA and cDNA Synthesis

Total RNA was isolated from different samples using the RNA-Solv^®^ reagent (Omega Bio-tek, Norcross, GA, USA) according to the manufacturer’s instructions. RNA concentrations were determined using a NanoDrop 2000 UV Spectrophotometer (Thermo Fisher Scientific, Cheshire, UK). The genomic DNA removed with (10× gDNA Remover Mix) and the first strand of cDNA was synthesized using a HiFiScript gDNA Removal cDNA Synthesis Kit (Cwbiotech, Taizhou, China) according to the manufacturer’s protocol, followed by storage at −80 °C until use.

### 2.3. Gene Cloning and Bioinformatics Analyses

A keyword-based screening of our RNAseq library (unpublished) yielded a short transcript homologue of the *CFSH*; this was validated by reverse transcription polymerase chain reaction (RT-PCR) and elongated by the 3′ and 5′ rapid amplification of cDNA ends (RACE) (Clontech, California, USA) using cDNA generated from muscle-extracted RNA. Table 1 shows the primers used in the cDNA clone. The following program was used for each PCR amplification: initial denaturation at 94 °C for 5 min, followed by 35 cycles of 94 °C for 30 s, 55 °C for 30 s and 72 °C for 90 s, with a final elongation at 72 °C for 10 min. All PCR products were analyzed using 1% agarose gel electrophoresis, then ligated with the pMD19-T vector (Takara, Kyoto, Japan) and transformed into competent *Escherichia coli* cells. After transformation, three positive clones were picked for sequencing (BGI Tech, Shenzhen, China). The amplified sequences from 3′ and 5′ RACE were assembled with the partial cDNA sequence using Vector NTI 10.0 software, and the open reading frame (ORF) was identified using ORF finder (http://www.ncbi.nlm.nih.gov/gorf/) (accessed on 22 January 2022). Predictions of the conserved domains were performed using the SMART program (http://smart.emblheidelberg.de/) (accessed on 22 January 2022). The 3D structure of the PtCFSH protein was predicted by the Alphafold2 web server (https://alphafold.ebi.ac.uk/) (accessed on 15 February 2022) and edited with the PyMOL Software (https://pymol.org/2/) (accessed on 27 February 2022). A multiple sequence alignment and phylogenic analysis was performed using ClustalW1.8 and MEGA7.0 (https://www.megasoftware.net/) (accessed on 28 February 2022).

### 2.4. Semiquantitative PCR

To detect the expression levels of *PtCFSH* in different tissues, primers PtCFSH-RTF/RTR (Table 1) were designed to generate a 212-bp fragment. Semiquantitative PCR was performed using β-actin as an internal reference to adjust the number of cDNA templates. Amplification was performed with the following program: denaturation at 94 °C for 5 min, followed by 35 cycles of 94 °C for 30 s, 55 °C for 30 s and 72 °C for 90 s, with a final elongation at 72 °C for 10 min. The products were assessed by electrophoresis on 1.5% agarose gel.

### 2.5. RNA Interference and Recombinant Protein Preparation and Injection

The double-strand RNA for PtCFSH, PtIAG, and green fluorescent protein (GFP) were synthesized as previously described [24]. The plasmid pGEX-4t-2 was digested with restriction endonucleases SmaI and XhoI in order to generate a linearized vector. Primers PtCFSH-GST-F/R with 21-bp (Table 1) extensions were designed and synthesized, which were homologous to the linearized vector 5′ and 3′ ends to generate the amplification products. The purified PCR product was inserted into the corresponding linearized vector by setting up an in-fusion cloning reaction using the ClonExpress II One Step Cloning Kit (Vazyme, Nanjing, China) and then transformed into *E. coli* cells. The confirmed recombinant plasmids were extracted using the E.Z.N.A. Plasmid Mini Kit I (Omega Bio-Tek) and then transformed into *E. coli* Rosetta-gami cells to express the rGST-CFSH protein. The vector pGEX-4t-2 as a control was also expressed as the GST tag protein (rGST). The recombinant proteins were purified using the GST-tag Purification Resin (Beyotime, Shanghai, China) and verified three times by SDS-PAGE.

For long-term treatments in vivo, the double-strand RNA of CFSH (dsCFSH) and the recombinant CFSH protein (rCFSH) were injected into crabs at 3 ug/g every three days for 20 days, with dsGFP and rGST serving as controls. The testes and AGs were collected to observe tissue changes. In the short-term RNAi treatment in vivo, dsCFSH, dsIAG, and dsGFP were all injected into different individuals at a dose of 3 ug/g dose, and samples were collected after 48 h. For the in vitro experiments, the desired eyestalks, AGs and testis tissues were all precultured in M199 medium for 1 h. Afterwards, dsCFSH and dsGFP were added at a dose of 3 μg/g dose [20] to Petri dishes (Jet Biofil, Guangzhou, China) containing the eyestalks and AGs, and then co-cultured at 26 °C for 8 h. Additionally, rCFSH and rGST were added to the dishes containing AGs or testes at doses of 10–6 M, respectively. Incubation was continued at 26 °C for 8 h. All samples were collected for qPCR assay and western blot analysis.

### 2.6. Quantitative Real-Time PCR

We determined the relative gene expression levels using quantitative real-time PCR (qPCR). The template cDNA was prepared as described above and then amplified with qPCR primers (Table 1). PCR was carried out using the SYBR^®^ Premix Ex Taq™ II Kit (Takara, Kyoto, Japan) according to the manufacturer’s instructions. PCR conditions were as follows: 95 °C for 2 min, followed by 40 cycles of 95 °C for 15 s and 56 °C for 20 s. Relative mRNA expression levels were normalized to β-actin mRNA expression [25] and calculated using the comparative Ct (2^−ΔΔCt^) method [26].

### 2.7. Western Blot Analysis

For the Western blot assay, the protein concentration of the testis was measured using the BCA Protein Assay Kit (Cwbiotech, Taizhou, China). For SDS-PAGE, 30 mg of protein from each sample was used, followed by electrophoretic transfer to a 0.45 βm PVDF membrane using a Mini Trans-Blot (Bio-Rad, California, USA). After blocking with Y-Tec Rapid Blocking Buffer (Yoche, Shanghai, China) at room temperature for 1 h, the membranes were incubated with Phospho-p44/42 mos/mitogen-activated protein kinase (MAPK) (Erk1/2) or p44/42 MAPK (Erk1/2) (Cell Signaling Technology, Danvers, MA, USA) monoclonal antibodies diluted to 1:1000 in (Y-Tec Stabilizing Antibody Diluent) (Yoche, Shanghai, China) overnight at 4 °C. The membranes were washed three times with TBST (20 mM Tris-HCl, 150 mM NaCl, and 0.05% Tween-20) and then incubated with β-Tubulin Rabbit IgG polyclonal antibody (CST) diluted to 1:2000 in Y-Tec Rapid Blocking Buffer (Yoche, Shanghai, China) at room temperature for 1 h. After washing three times with TBST for 10 min each, the membrane was incubated in a Western Bright ECL substrate (Advansta, California, USA) before exposure to ChemiScope 6000 (CLiNX, Shanghai, China). The protein bands were quantified using the ChemiScope figure software package, and the results were derived from the statistical analysis of three independent experiments.

### 2.8. Measurement of the Level of cAMP, cGMP, and NOS in Testis

The testis was collected at 5, 15 min and 6 h after rCFSH (10^−6^ M) and forskolin (10^−5^ M) treatment (three replicates per treatment). The cAMP levels and the cGMP levels of the sampled testis at 5 and 15 min were measured using the cAMP ELISA kit (Nanjing Jiancheng Bioengineering Institute of China, Nanjing, China) and cGMP ELISA kit (Nanjing Jiancheng Bioengineering Institute of China, Nanjing, China), respectively. Samples collected after 6 h of treatment were used to detect changes in nitric oxide synthase (NOS) levels using a NOS-typed assay kit (Nanjing Jiancheng Bioengineering Institute of China, Nanjing, China).

### 2.9. Histological Observation of the Testis of RNAi and Recombinant Protein Injection

At the end of the long-term RNAi and recombinant protein injection experimental period, crabs from four groups with a body width of 12.2–14.6 cm were collected. Testis were dissected from the individuals, cut into 3 mm^3^ pieces, and fixed in PFS for 24 h at 4 °C. After dehydration in gradient ethanol, they were embedded in paraffin. Paraffin-embedded AGs and testis tissues were sectioned into slices of 5–7 μm. The sections were then stained with H&E and observed under a Nikon Eclipse 80i microscope (Nikon, Tokyo, Japan).

### 2.10. Conceptual Diagrams of Hypothetical Pathways

This hypothetical pathway was constructed with reference to the classical insulin pathway and the general neuropeptide signaling pathway [27]. In this paper, we complete the hypothetical pathway by figdraw.

### 2.11. Statistical Analysis

All data are expressed as mean ± SEM and were subjected to a one-way analysis of variance (ANOVA), followed by Student’s *t*-test or Tukey’s multiple-group comparison test. The data were analyzed using SPSS 24.0 software. Significant differences were accepted at *p* < 0.05. Asterisk denotes statistically significant differences: * *p* < 0.05; ** *p* < 0.01; *** *p* < 0.001.

## 3. Results

### 3.1. Molecular Characterization and Tissue Distribution of PtCFSH

**Figure 1:**
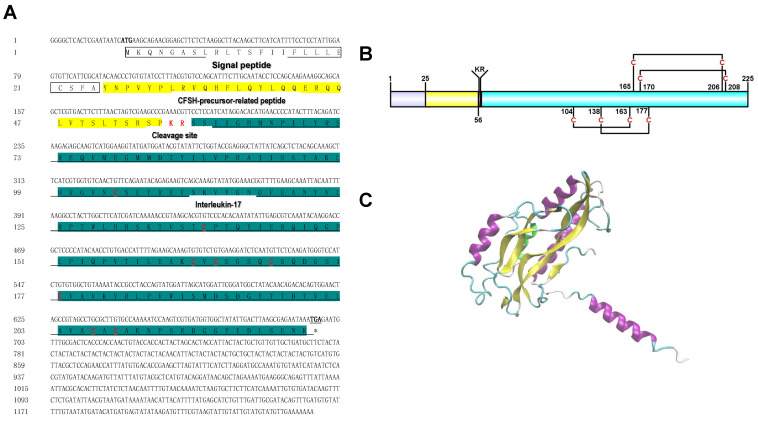
The molecular characterization of PtCFSH. (**A**) Nucleotide and amino acid sequences of PtCFSH. Bolded markers are the nucleotide and amino acid sequences encoding the start codon (ATG) and * indicates termination codon (TGA). Predicted signal peptide sequences and the CFSH-precursor-related peptides are shown in white and yellow boxes, respectively. The cleavage site “KR” is marked in red. The major domain interleukin-17 is marked with a green box and underlined, and the 8 cysteines are marked in red. (**B**) Structure description of PtCFSH. The ORF contains four different structures, the purple box is the signal peptide, the yellow box is the precursor-related peptide, the red box is the cleavage site “KR”, and the blue box is the interleukin-17domain containing four disulfide bonds. (**C**) The putative 3D structure of mature PtCFSH predicted by the Alphafold2.

We obtained a 1241 bp *PtCFSH* cDNA (Genbank accession number: ON929327) comprising a 19 bp 5′-untranslated region (UTR), 678 bp ORF, and 542 bp 3′-UTR with a poly (A) tail (Figure 1). A sequence analysis of the 678 bp ORF revealed 225 amino acids with a 24 aa signal peptide, 31 aa CFSH-precursor-related peptides, a 2 aa cleavage site and a 166 aa interleukin-17 domain containing eight cysteines, forming four disulfide bonds (Figure 1A,B). The three-dimensional structure of the CFSH was composed of α-helix and connected random coils, as shown by the Alphafold2 results (Figure 1C).

**Figure 2:**
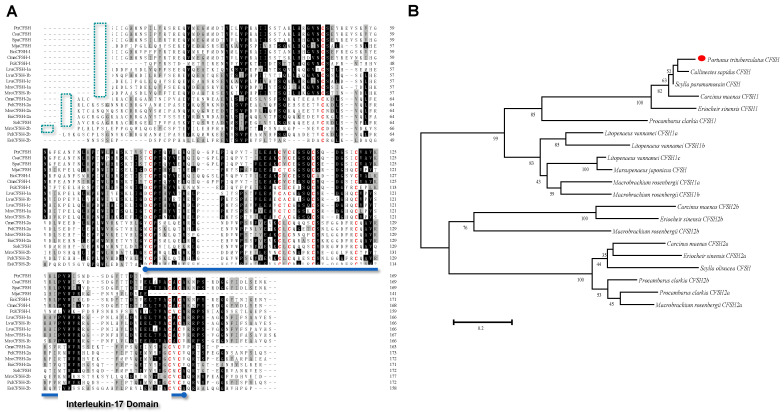
Multiple sequence alignment and phylogenetic tree of the CFSH mature peptide in decapoda. (**A**) Multiple amino acid sequence alignment of insulin family peptides. Most of the CFSH sequences were borrowed from previous works by [21]; other sequences were shown in Table 2. Cleavage site “KR” is marked with a green box. The eight cysteines are marked in red and the interleukin-17 domain is marked with a blue underline. (**B**) Phylogenetic tree of CFSHs in crustaceans. Phylogenetic analysis was conducted by the neighbor-joining method in MEGA7. The percentage of replicate trees in which the associated taxa clustered together in the bootstrap test (1000 replicates) was shown next to the branches. PtCFSH was indicated by a red dot.

It was shown by multiple sequence alignments of PtCFSH with other crustacean CFSHs that the predicted PtCFSH protein shares significant identities with other CFSHs in the cleavage site “KR”, including “Cysteine” in the interleukin-17 domain (Figure 2A). Furthermore, it was revealed in phylogenetic analysis that the PtCFSH was more closely related to the other CFSH, while it was divided into two branches with subtype CFSH2 (Figure 2B). The transcript levels of *PtCFSH* were measured in different tissues obtained from the adult male. It was demonstrated by these results that the *PtCFSH* transcripts were mainly expressed in the eyestalk, with a slight expression in the brain and testis (Supplementary Figure S1).

### 3.2. Effects of PtCFSH on AG

**Figure 3:**
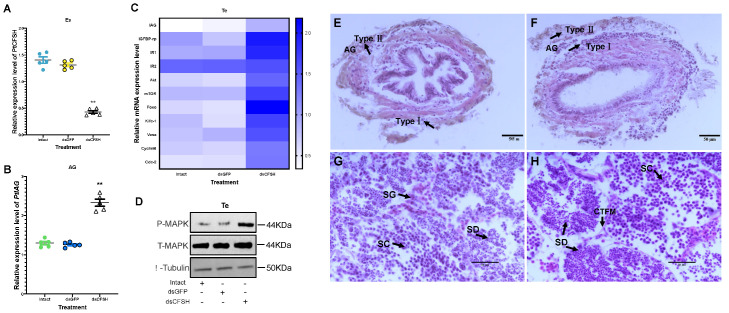
Effects of long-term dsPtCFSH injection on gene and protein expression of *P. trituberculatus*. (**A**) The efficacy of gene knockdown in the long-term PtCFSH silencing experiment was evaluated by qRT-PCR in the eyestalk. ** indicates acceptance *p* < 0.01. (**B**) Effect of dsPtCFSH on *PtIAG* in AG tissue. The expression levels of *PtIAG* were examined in the intact group and after the in vivo injection of dsGFP and dsPtCFSH. ** indicates acceptance *p* < 0.01. (**C**) Effect of dsPtCFSH on a series of genes in the testis. The heatmap levels of *PtIAG*, *PtIGFBP*-rp, *PtIR1*, *PtIR2*, *PtAkt*, *PtmTOR*, *PtFoxo*, *PtKifc*-1, *PtVasa*, *PtCyclinB*, *PtCdc*-2 were examined in the intact group and after the in vivo injection of dsGFP and dsCFSH. Different colors of the same tested gene between the experimental and control groups indicated significant differences. The darker the color in the heat map, the higher the relative expression of the tested genes and vice versa. (**D**) Effect of dsPtCFSH on phosphorylated-MAPK in testis. Phosphorylated MAPK, total MAPK and β-tubulin were detected in the intact group, dsGFP and dsPtCFSH injection groups, with T-MAPK and tubulin serving as internal references. (**E**) I Histological sectioning and H&E staining of AG after dsGFP injection. The arrows indicate type I and II AG cells, and the scale bar is marked in the lower right corner. (**F**) Histological sectioning and H&E staining of AG after dsPtCFSH injection. The arrows indicate type I and II AG cells, and the scale bar is marked in the lower right corner. (**G**) Histological sectioning and H&E staining of the testis after dsGFP injection. The arrows point to the spermatogonia (SG), spermatocytes (SC) and spermatid (SD), respectively. The scale bar is shown in the lower right corner. (**H**) Histological sectioning and H&E staining of testis after dsPtCFSH injection. The arrows point to the spermatogonia (SG), spermatocytes (SC) and spermatid (SD), and connective tissue fibers muscle (CTFM), respectively. The scale bar is shown in the lower right corner. Vertical bars represent means ± SEM (n = 5). Significant differences among stages are indicated by different letter labels (one-way ANOVA followed by post hoc Tukey’s multiple group comparison *p* < 0.05).

**Figure 4:**
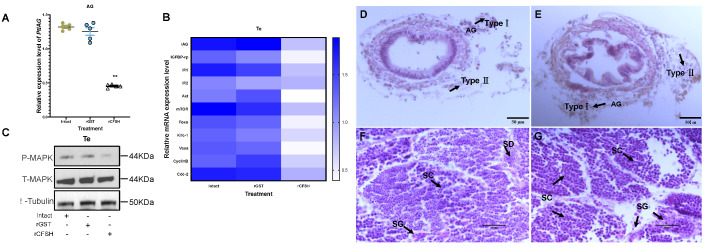
Effects of long-term rPtCFSH injection on gene and protein expression of *P. trituberculatus*. (**A**) Effect of rPtCFSH on *PtIAG* in AG tissue. The expression levels of *PtIAG* were examined in the intact group and after the in vivo injection of rGST and rPtCFSH. ** indicates acceptance *p* < 0.01. (**B**) Effect of rPtCFSH on a series of genes in the testis. The heatmap levels of *PtIAG*, *PtIGFBP-rp*, *PtIR1*, *PtIR2*, *PtAkt*, *PtmTOR*, *PtFoxo*, *PtKifc*-1, *PtVasa*, *PtCyclinB*, *PtCdc*-2 were examined in the intact group and after the in vivo injection of rGST and rPtCFSH. Different colors of the same tested gene between the experimental and control groups indicated significant differences. The darker the color in the heat map, the higher the relative expression of the tested genes and vice versa. (**C**) Effect of rPtCFSH on phosphorylated-MAPK in testis. Phosphorylated MAPK, total MAPK and β-tubulin were detected in the intact group, rGST and rCFSH injection groups, with T-MAPK and tubulin serving as internal references. (**D**) Histological sectioning and H&E staining of AG after rGST injection. The arrows indicate type I and II AG cells, and the scale bar is marked in the lower right corner. (**E**) Histological sectioning and H&E staining of AG after rPtCFSH injection. The arrows indicate type I and II AG cells, and the scale bar is marked in the lower right corner. (**F**) Histological sectioning and H&E staining of the testis after rGST injection. The arrows point to the spermatogonia (SG), spermatocytes (SC) and spermatid (SD), respectively. The scale bar is shown in the lower right corner. (**G**) Histological sectioning and H&E staining of testis after rPtCFSH injection. The arrows point to the spermatogonia (SG), spermatocytes (SC) and spermatid (SD), respectively. The scale bar is shown in the lower right corner. Vertical bars represent means ± SEM (n = 5). Significant differences among stages are indicated by different letter labels (one-way ANOVA followed by post hoc Tukey’s multiple group comparison *p* < 0.05).

**Figure 5:**
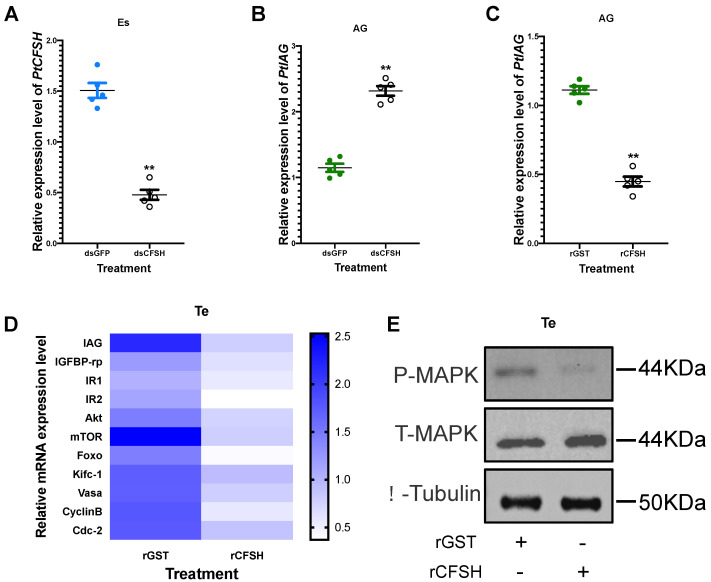
Effects of rPtCFSH and dsPtCFSH in vitro treatment on gene and protein expression of *P. trituberculatus.* (**A**) Interference efficiency of in vitro co-culture of the eyestalk and AG and treated with dsPtCFSH. The mRNA transcription of PtCFSH was significantly reduced in the eyestalk compared to dsGFP, with an interference efficiency of 71.4%. ** indicates acceptance *p* < 0.01. (**B**) Effect of in vitro co-culture of the eyestalk and AG and treated with dsPtCFSH on *PtIAG*. The expression of *PtIAG* was remarkably increased in the AG. ** indicates acceptance *p* < 0.01. (**C**) Effects of rPtCFSH in vitro treatment on *PtIAG*. qPCR results showed that *IAG* in AG was down-regulated dramatically after being subjected to *PtCFSH*. ** indicates acceptance *p* < 0.01. (**D**) Heat map of gene expression levels in the testis after rPtCFSH in vitro treatment. Different colors of the same tested gene between the experimental and control groups indicated significant differences. The darker the color in the heat map, the higher the relative expression of the tested genes and vice versa. (**E**) Changes in phosphorylated-MAPK after rPtCFSH in vitro treatment of testis. Total-MAPK, β-tubulin as internal reference protein. The scale bar is shown in the lower right corner. Vertical bars represent means ± SEM (n = 5). Significant differences among stages are indicated by different letter labels (one-way ANOVA followed by post hoc Tukey’s multiple group comparison *p* < 0.05).

In order to clarify the inhibitory effect of the CFSH on the AG, dsPtCFSH and rPtCFSH were used to conduct the in vivo and in vitro experiments (Figure 3, Figure 4 and Figure 5). Treatments with dsPtCFSH led to a significant down-regulation of *PtCFSH* gene expression in the eyestalk, both in vivo and in vitro, showing a high interference efficiency (Figure 3A and Figure 5A). The long-term silencing in PtCFSH in vivo caused an increase in the ratio of type I AG cells and a decrease in type II AG cells, while the injection of rPtCFSH showed an opposite effect (Figure 3E,F and 4D,E). The types of AG cells were classified according to Sroyraya et al. [28]. Briefly, the type I AG cells have a small globular nucleus containing mainly heterochromatin, while the type II cells are vacuolated with a large cell diameter, containing mostly euchromatin and prominent ribosomes. It was found that the type I cells synthesize IAG in a greater amount than the type II cells [28]. Consistent with the change in the IAG-producing cells, qPCR analysis showed that the *PtIAG* expression could be reduced by rPtCFSH injection (Figure 4A), while induced by *PtCFSH* silencing (Figure 3B). Similar results were also observed in in vitro assays (Figure 5B,C), further confirming the inhibitory role of the CFSH on *IAG* expression.

### 3.3. Effects of PtCFSH on Testis

To investigate the function of *PtCFSH* on the testicular development of *P. trituberculatus*, the histological features of the testis and the expression of spermatogenesis-related genes were evaluated after the in vivo *PtCFSH* silencing and rPtCFSH injection. It was shown that the treatment of rPtCFSH delayed the further conversion of spermatocytes to spermatids, when compared to the controls (Figure 4F,G). In contrast, a gradual transformation of spermatocytes to spermatids in the testis was observed in the PtCFSH silencing group (Figure 3G,H), in parallel with a gradual production of CTFM (Figure 3H). Meanwhile, the mRNA expression of spermatogenesis-related genes in the testis, such as the kinesin-like protein (*Kifc-1*), ATP-dependent RNA helicase (*Vasa*), *CyclinB* and cyclin-dependent kinase-2 (*Cdc-2*) [29], was reduced by rPtCFSH injection and induced by *PtCFSH* silencing (Figure 3C and Figure 4B). The combined results suggest that *PtCFSH* negatively regulates the spermatogenesis of *P. trituberculatus*.

To find out whether the negative regulatory effect of the CFSH is achieved through IAG, the changes in expression levels of several classical insulin pathway genes, including *IAG* itself, insulin-like growth factor banding protein-related peptide (*IGFBP-rp*), insulin-like hormone receptor 1 (*IR1*), *IR2*, protein kinase B (*Akt*), mammalian target of rapamycin (*mTOR*), and forkhead transcription factors (*Foxo*) [30,31], were further determined in the testis in the two treatments. In addition, since the insulin pathway activates the phosphorylation of MAPK [32], the change in the phosphorylated MAPK levels were also examined using Western blotting. As expected, the expression of insulin pathway genes and phosphorylated MAPK levels were reduced by rPtCFSH injection and induced by *PtCFSH* silencing (Figure 3C,D and Figure 4B,C). Therefore, it was speculated that the CFSH can inhibit the production of IAG, which, in turn, affects the testicular development.

Furthermore, in vitro experiments were conducted to explore whether the CFSH had a direct regulatory effect on the testis. It was found that the addition of rPtCFSH into the testis explants also led to a decrease in spermatogenesis-related gene expression and the MAPK phosphorylation level, which means that the CFSH could act directly on the testis (Figure 5D,E). Meanwhile, it was also observed that the expression levels of *IAG* and insulin pathway genes in the testis were down-regulated (Figure 5D).

### 3.4. Validation of the CFSH–IAG–Testis Endocrine Axis

**Figure 6:**
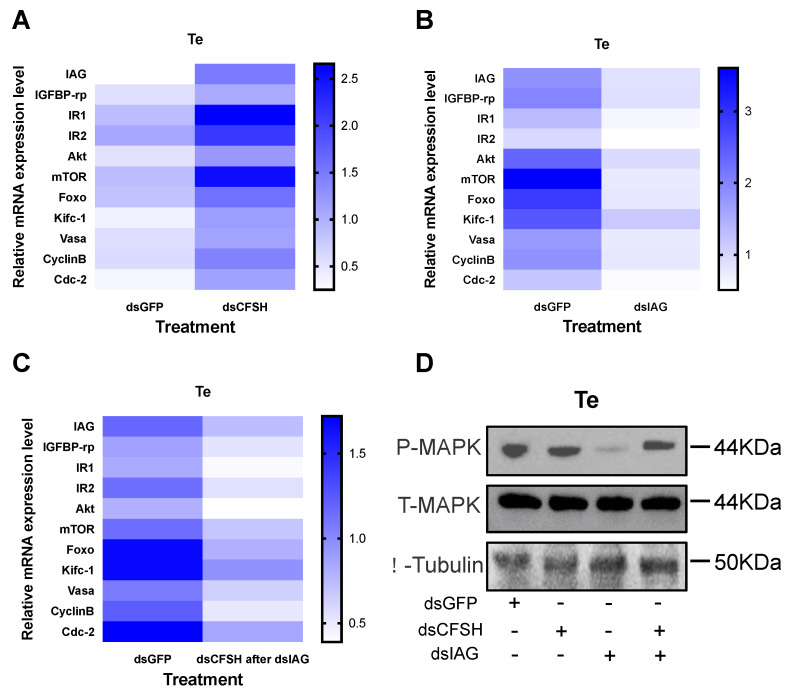
Effects of short-term in vivo injection of dsPtCFSH, dsPtIAG on related genes and proteins expression. (**A**) Heat map expression levels of related genes after injection of dsPtCFSH, dsGFP. Different colors of the same tested gene between the experimental and control groups indicated significant differences. The darker the color in the heat map, the higher the relative expression of the tested genes and vice versa. (**B**) Heat map expression levels of related genes after injection of dsPtIAG, dsGFP. Different colors of the same tested gene between the experimental and control groups indicated significant differences. The darker the color in the heat map, the higher the relative expression of the tested genes and vice versa. (**C**) Heat map expression levels of related genes after injection of dsPtIAG followed by dsPtCFSH, dsGFP. Different colors of the same tested gene between the experimental and control groups indicated significant differences. The darker the color in the heat map, the higher the relative expression of the tested genes and vice versa. (**D**) Changes in phosphorylated-MAPK expression levels after injection of dsPtIAG followed by dsPtCFSH, dsPtCFSH, and dsGFP. Total-MAPK, β-tubulin as internal reference proteins. The scale bar is shown in the lower right corner. Vertical bars represent means ± SEM (n = 5). Significant differences among stages are indicated by different letter labels (one-way ANOVA followed by post hoc Tukey’s multiple group comparison *p* < 0.05).

A short-term RNAi experiment was performed to verify the existence of the CFSH–IAG–testis endocrine axis. Similar changes in the levels of gene expression and MAPK phosphorylation were observed in short-term injection with dsPtCFSH, when compared to those in long-term treatment (Figure 6A,D). Opposite effects were found in the dsPtIAG injection group (Figure 6B,D), indicating the stimulatory role of *IAG* in testicular development. To confirm the central role of IAG in this endocrine axis, a mixed injection of dsPtCFSH and dsPtIAG was further performed, and the results were similar to those of the dsPtIAG injection alone (Figure 6C,D). It can be understood that the regulatory effect of dsPtCFSH on the testicular development was blocked after the injection of dsPtIAG, which indicated the existence of the CFSH–IAG–testis endocrine axis.

### 3.5. Possible Signaling Mechanisms of PtCFSH

**Figure 7:**
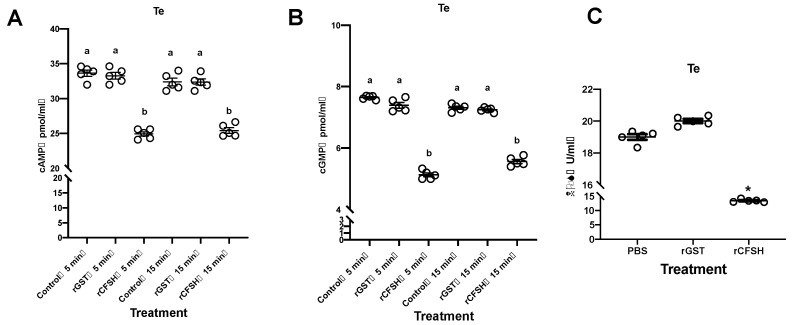
Changes in cAMP, cGMP, and NOS levels were detected after the rPtCFSH isolated culture of testis. (**A**) Changes in cAMP levels in the testis after rPtCFSH treatment compared to rGST, PBS group. Groups with the same letter do not differ significantly, and different letters indicate significant differences. (**B**) Changes in cGMP levels in the testis after rPtCFSH treatment compared to rGST, PBS group. Groups with the same letter do not differ significantly, and different letters indicate significant differences. (**C**) Changes in NOS levels in the testis after rPtCFSH treatment compared to rGST, PBS group. The scale bar is shown in the lower right corner. * indicates acceptance 0.01 < *p* <0.05. Vertical bars represent means ± SEM (n = 5). Significant differences among stages are indicated by different letter labels (one-way ANOVA followed by post hoc Tukey’s multiple group comparison *p <* 0.05).

Most neuropeptides use the G protein-coupled receptor (GPCR) as their membrane receptor, and the signaling system needs the participation of second messengers [27]. To explore which second messengers are involved in the signal transduction of PtCFSH, the levels of cAMP and cGMP, as well as the activity of nitric oxide synthetase (NOS), were determined in in vitro testis experiments. Remarkable down-regulation at 5 min and 15 min after adding rPtCFSH was shown by both cAMP and cGMP levels compared to the control group (Figure 7A,B). In addition, the NOS enzymatic activity was significantly diminished after adding rPtCFSH, suggesting a decrease in the NO level (Figure 7C).

## 4. Discussion

The CFSH is a primary hormone involved in developing relevant phenotypes in female crustaceans [16,22,23,33]. However, the regulatory mechanisms of CFSH in male crustaceans have been under investigated. The full-length *CFSH* gene sequence of *P. trituberculatus*, encoding 225 amino acids, including the signal peptide, a cleavage KR site, and an IL-7 structural domain containing eight cysteines, was investigated in this study. It was shown that it has the same structural organization as type I CFSHs. Multiple sequence comparisons and a phylogenetic tree analysis supported that PtCFSH belongs to type I CFSHs (Figure 1 and Figure 2). Most type I CFSHs contain eight cysteines [17,18,19,20,21,22,34,35], which play a role in forming the disulfide bonds necessary for their normal biological function [21,36,37]. In crustaceans, it has been reported in various species that it is exclusively expressed in the eyestalk, such as *CsaCFSH*, *SpCFSH*, and *LvCFSH1a*, which have been shown to stimulate female sexual differentiation [16,22,33]. In contrast, *MrCFSH1a/b* and *MajCFSH* were detected in several distinct tissues. Similar results were also observed in the present study, showing that, in parallel to the strong signal detected in the eyestalk, *PtCFSH* also has a slight signal within the testicular tissue (Supplementary Material). Thus, the *CFSH* may play a more extensive role in decapod crustaceans [19,21].

In the present study, the physiological role of *PtCFSH* in male *P. trituberculatus* was investigated in a series of in vivo and in vitro experiments, based on the dsPtCFSH RNA silencing and rPtCFSH treatment. Consistent with the reported negative regulation of IAG by CFSH [22,23], *PtCFSH* silencing and rPtCFSH treatment both suggested that PtCFSH can significantly inhibit the expression level of *PtIAG* in vitro and in vitro. Furthermore, the histological study on the AG showed that PtCFSH had a negative effect on the number of IAG-producing cells. This would be reminiscent of the bilateral eyestalk ablation of the *P. pelagicus* that caused an increase in the cells producing IAG [28]. In *Macrobrachium nipponense*, RNAi in several eyestalk neuropeptides revealed that the gonad-inhibiting hormone (GIH) and molt-inhibiting hormone (MIH) had an inhibitory effect on IAG expression [15]. Our results confirmed that the CFSH is also a neuropeptide with IAG inhibition.

It was revealed in our in vivo studies that rPtCFSH injection could reduce the conversion of spermatocytes to spermatids (Figure 4F,G), while this process was stimulated when dsCFSH was silenced (Figure 3G,H). In addition, the expression levels of spermatogenesis-related genes, such as *kifc-1*, *vasa*, *cyclinB*, and *cdc-2* [38,39], changed accordingly in two treatments, suggesting that PtCFSH may be involved in testis development and spermatogenesis in *P. trituberculatus*. The effects of PtCFSH on testis might be achieved through IAG, since several classical insulin pathway genes, including *IAG* itself, insulin-like growth factor binding protein-related peptide (*IGFBP-rp*), insulin-like hormone receptor 1 (*IR1*), *IR2*, protein kinase B (*Akt*), mammalian target of rapamycin (*mTOR*), and forkhead transcription factors (*Foxo*) [30,31,40,41], were also induced by *PtCFSH* silencing and simultaneously reduced by rPtCFSH. As one of the two major intracellular pathways in the conserved insulin signaling system, the MAPK pathway mainly controls development and reproduction [42]. Therefore, the change in testicular MAPK phosphorylation levels in PtCFSH-based manipulations (Figure 3D and Figure 4C) might give further evidence that IAG is involved in the CFSH-mediated testicular development.

To confirm the existence of the CFSH–IAG–testis endocrine axis, a short-term injection of dsPtIAG was further performed. It was found that this treatment reduced the levels of gene expression and MAPK phosphorylation. Given the inhibitory effect of PtCFSH on *IAG* expression, dsPtCFSH was also injected in the short-term experiments, with the purpose of causing a transient increase in *PtIAG* expression; this obtained opposite effects to the *PtIAG* silencing. In fact, the short-term silencing in *PtCFSH* confirms the regulatory relationship between *PtCFSH* with *PtIAG* and related genes, which had been revealed in the long-term experiments. The co-injection of both dsPtCFSH and dsPtIAG showed similar effects on the testis to the single injection of dsPtIAG; it could be understood that the regulatory effect of dsPtCFSH on the testis was blocked after the injection of dsPtIAG, demonstrating the central role of IAG in connecting the CFSH and testis.

In addition to the indirect effects of PtCFSH on the testis, the in vitro treatment of rPtCFSH on the testis explants suggested that the PtCFSH was able to act directly on the testis. Interestingly, however, this direct effect might also be related to the IAG. Our results showed that rPtCFSH can also inhibit the levels of *PtIAG* and insulin pathway gene expression in the testis, as well as the MAPK phosphorylation. Although the presence of IAG in testis has been less reported, it was revealed by our previous study that *PtIAG* is expressed in the testis of *P. trituberculatus* [43]. Since the paracrine/autocrine is considered to be an important mechanism of crustacean hormonal regulation [44,45,46], the testis-derived IAG might also be involved in the regulation of testicular development. Therefore, the inhibitory effect of the CFSH on IAG expression might not be restricted to the AG, but might also occur in other tissues. One circumstantial piece of evidence may come from studies of the CFSH in females, finding that the CFSH can inhibit the expression of IAG in the ovaries [22].

To exert its physiological functions, whether on the AG or the testis, the CFSH must have its own set of signaling mechanisms. As a neuropeptide, GPCR is a primary candidate for the CFSH receptor [27]. However, since numerous types of GPCRs exist [33], the receptors for the CFSH has not been firmly validated. Among the signaling pathways regulated by GPCRs, the second messenger is particularly important, and cAMP, cGMP, and NO are the most common ones [47]. Therefore, we examined the level changes in these second messengers in testis explants after rPtCFSH treatment. It was found that the level of cAMP and cGMP, as well as the NOS activity, were all down-regulated by rPtCFSH. This would be reminiscent of the signaling system deduced for MIH, which is constituted of a “triggering phase” involving cAMP/Ca^2+^ pathway, a “summation phase” involving the NO/cGMP pathway, and the calmodulin (CaM), which links the two phases by activating NOS [27]. It is likely that the CFSH uses a similar system for its signal transduction. Furthermore, these results also imply that the PtCFSH might act on the testis, independent of IAG.

## 5. Conclusions

**Figure 8:**
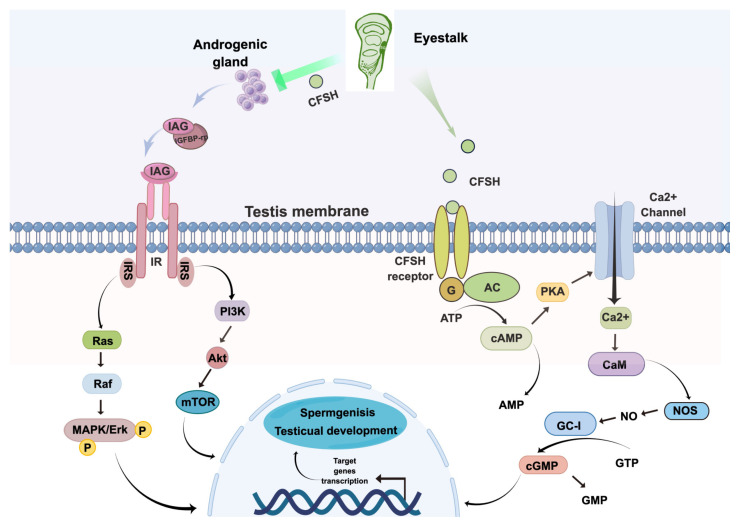
Hypothetical pathway for the mode of the CFSH action in *P. trituberculatus*. There may be different pathways for CFSH. One is acting on the testis through the classical insulin pathway and the other is acting directly as a neuropeptide to complete the signal transduction. IAG: insulin-like androgenic gland hormone; IGFBP-rp: insulin growth factor binding protein-related protein; IR: insulin-like hormone receptor; IRS: Insulin receptor substrate; G: G protein; AC: activation of adenylyl cyclase; GC-I: NO-sensitive guanylyl cyclase.

In conclusion, the *P. trituberculatus* CFSH was identified, cloned and expressed in the recombinant form. The physiological functions of PtCFSH on the AG and testis were investigated using the RNAi and recombinant protein injection in a series of in vivo and in vitro experiments. It was confirmed that PtCFSH can negatively regulate the spermatogenesis and testicular development via a CFSH–IAG–testis endocrine axis. PtCFSH may also act directly on the testis, dependent or independent of IAG. The signaling system of PtCFSH involved the participation of cAMP, cGMP, and NO, which resembles the MIH signaling pathway. In order to summarize, a hypothetical pathway for the mode of the CFSH action in *P. trituberculatus* was constructed by figdraw (Figure 8).

## Figures and Tables

**Table 1:** Primers used in this study.

Name	Sequence (5′-3′)	PCR Objective
PtCFSH-F1	CGTCAAATACAAGGACCGCTC	3′ RACE
PtCFSH-F2	CAATGTTCTCAAGATGGGTCC	3′ RACE
PtCFSH-R1	CCTCGCAGTCCAGAACCAA	5′ RACE
PtCFSH-R2	GTGATAGAGGGCGTGGG	5′ RACE
PtCFSH-F	TCCTATTGGAGTGTTCATTCGC	cDNA clone
PtCFSH-R	GTGGTGGTACAGTTGGTGGG	cDNA clone
PtCFSH-RT-F	ACCGCCTACCAGTATGGATTAG	RT-PCR
PtCFSH-RT-R	GCATCAGCAACAACAGCAGTA	RT-PCR
5′outer	CTAATACGACTCACTATAGGGC	5′ RACE
5′inner	AAGCAGTGGTATCAACGCAGAGT	5′ RACE
3′inner	TCCACTAGTGATTTCACTATAGG	3′ RACE
3′outer	CTAATACGACTCACTATAGGGC	3′ RACE
AP	TACCGTCGTTCCACTAGTGATTTCACTATAGG(T)17	3′ RACE
dsGFP-F	TAATACGACTCACTATAGGGCGACGTAAACGGCCACAAGT	RNAi
dsGFP-R	TAATACGACTCACTATAGGGCTTGTACAGCTCGTCCATGC	RNAi
dsPtCFSH-F	TAATACGACTCACTATAGGGAGATCCTATTGGAGTGTTCATTCG	RNAi
dsPtCFSH-R	TAATACGACTCACTATAGGGAGATACAGTTGGTGGGTGAGTCG	RNAi
dsPtIAG-F	TAATACGACTCACTATAGGGAAACGAAGACCCAATGCTACC	RNAi
dsPtIAG-R	TAATACGACTCACTATAGGGTTACTGCCTATTTCGGGAAGC	RNAi
PtCFSH-QF	GTATTTCATCTTAGGATGCCAA	qRT-PCR
PtCFSH-QR	TAAACTCTGCCCTTCATTTTCT	qRT-PCR
β-actin-QF	CGAAACCTTCAACACTCCCG	qRT-PCR
β-actin-QR	GATAGCGTGAGGAAGGGCATA	qRT-PCR
PtIGFBP-rp-QF	TTACCACTATTGACGGCACCT	qRT-PCR
PtIGFBP-rp-QR	TCATTATC TGTACCCATCCTGTT	qRT-PCR
PtIAG-QF	TCTTATTAGCGACTTCTCCG	qRT-PCR
PtIAG-QR	CCTCTGTCCCTCGTTTATGT	qRT-PCR
PtIR1-QF	CTGATGCGTTTGTCGTATTT	qRT-PCR
PtIR1-QR	GAAGCGTGGTGCCTATTT	qRT-PCR
PtIR2-QF	ACCAGCTAGTGGGAACCG	qRT-PCR
PtIR2-QR	GGGAGGGACTCTTTGACG	qRT-PCR
PtAkt-QF	CTCAACCAGGAACGCTTCTTC	qRT-PCR
PtAkt-QR	TGTGTCCATCAGCATCCAGTAA	qRT-PCR
PtmTOR-QF	TCTCCTGGCTGTTGCTGTC	qRT-PCR
PtmTOR-QR	GCTTCTTGCTTGGTGTATCCTT	qRT-PCR
PtAkt-QF	CTCAACCAGGAACGCTTCTTC	qRT-PCR
PtAkt-QR	TGTGTCCATCAGCATCCAGTAA	qRT-PCR
Ptcdc2-QF	CCGTCAAGCAGATGGACAGTG	qRT-PCR
Ptcdc2-QR	CCAGGTCGTCAAAGTAAGGGTG	qRT-PCR
PtCyclinB-QF	ATGTGCCACTACAAGGCGTCT	qRT-PCR
PtCyclinB-QR	ATCAGCGTGTCATTCCAATCC	qRT-PCR
PtFoxo-QF	CGGAGGTGAAGCACATCAAC	qRT-PCR
PtFoxo-QR	TCATTGGTGGAGGCAGAGTG	qRT-PCR
PtKifc1-QF	TCCAATCGCCATCTACCTCAG	qRT-PCR
PtKifc1-QR	CGTCTTCAGCATCTCCAGAATG	qRT-PCR
PtVasa-QF	GCTTGCCATCCAGATATTCCAT	qRT-PCR
PtVasa-QR	TGCTCCTTCATACGCCTCAA	qRT-PCR
PtCFSH-GST-SmaI-F	ggatccccaggaattcccgggATGAAGCAGAACGGAGCTTCTC	In-Fusion clone
PtCFSH-GST-XhoI-R	gtcacgatgcggccgctcgagTCATTTATTCTCGCTTAAGTCAATATAGC	In-Fusion clone

**Table 2:** List of species used in multiple sequence comparison phylogenetic analysis.

Species	Protein Name	GenBank Accession Number
*Portunus trituberculatus*	CFSH	ON929327
*Scylla paramamosain*	CFSH	MN938502.1
*Callinectes sapidus*	CFSH	GU016328.1
*Carcinus maenas*	CFSH-1	AEI72264.1
*Marsupenaeus japonicus*	CFSH	BBA53799.1

## Data Availability

*PtCFSH* nucleic acid sequence has been uploaded to NCBI (GenBank: ON929327).

## Supplementary Materials

The following supporting information can be downloaded at: https://www.mdpi.com/article/10.3390/ani13040690/s1, Figure S1: Detection of PtCFSH expression in tissues by RT-PCR; Figure S2: Prokaryotic expression and purification of PtCFSH and empty vector; Figure S3: Phosphorylated-MAPK was detected in the testis after long-term in vivo injection of rPtCFSH; Figure S4: Phosphorylated-MAPK was detected in testis after long-term in vivo injection of dsPtCFSH, dsGFP; Figure S5: Phosphorylated-MAPK was detected in the testis after incubation of testis in vitro and addition of rPtCFSH and rGST, respectively; Figure S6: Phosphorylated-MAPK was detected in testis after short-term in vivo injection of dsCFSH, dsIAG, dsIAG followed by dsCFSH, and dsGFP, respectively; Table S1: Primers used in this study; Table S2: List of species used in phylogenetic analyses and multiple sequence comparisons.
