# Supplementary material for: Putative Role of CFSH in the Eyestalk-AG-Testicular Endocrine Axis of the Swimming Crab Portunus trituberculatus
Source: Animals (Basel). 2023 Feb 16;13(4):690. doi: 10.3390/ani13040690 (PMC9952137; doi:10.3390/ani13040690)
Supplement: Supplementary file 1 [file animals-13-00690-s001.zip › animals-2194698-supplementary.pdf]

Supplemental Materials

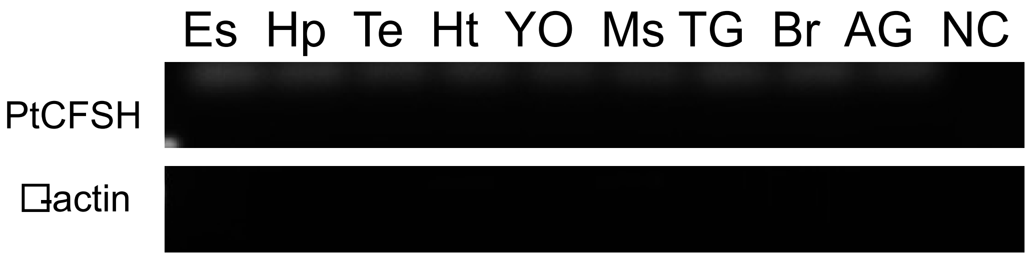

Supplemental Figure S1. Detection of PtCFSH expression in tissues by RT-PCR. β-actin as internal reference. Es: eyestalk; Hp: Hepatopancreas; Te:testis; Ht: heart; YO: Y organ; Ms: muscle; TG: Thoracic ganglia; Br: Brain; AG: androgenic gland; NC: negative control.

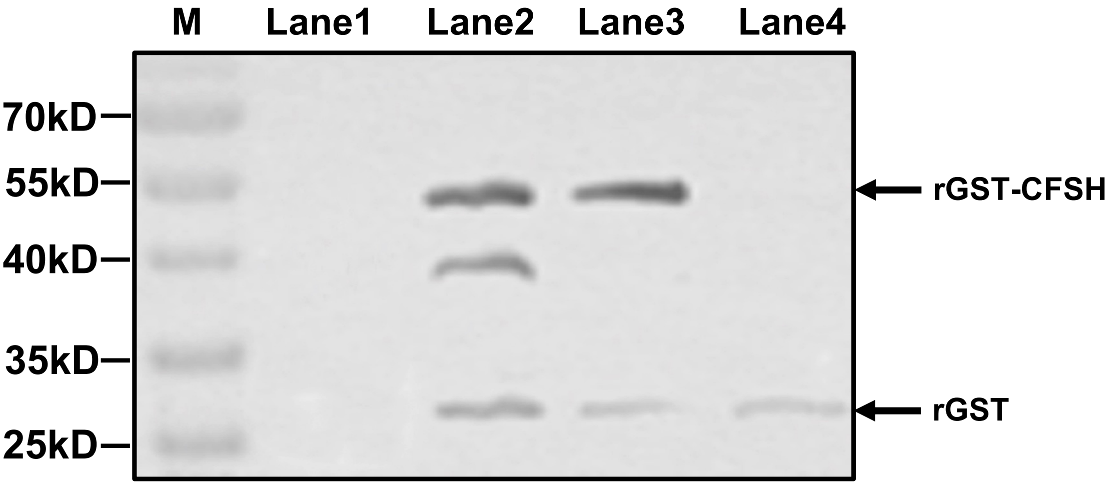

Supplemental Figure S2. Prokaryotic expression and purification of PtCFSH and empty vector. Lane1: Pre-induction; Lane2: Post-induction; Lane3: Post-purified rGST-PtCFSH; Lane4: Post-purified rGST; Arrows indicate the size of protein molecules.

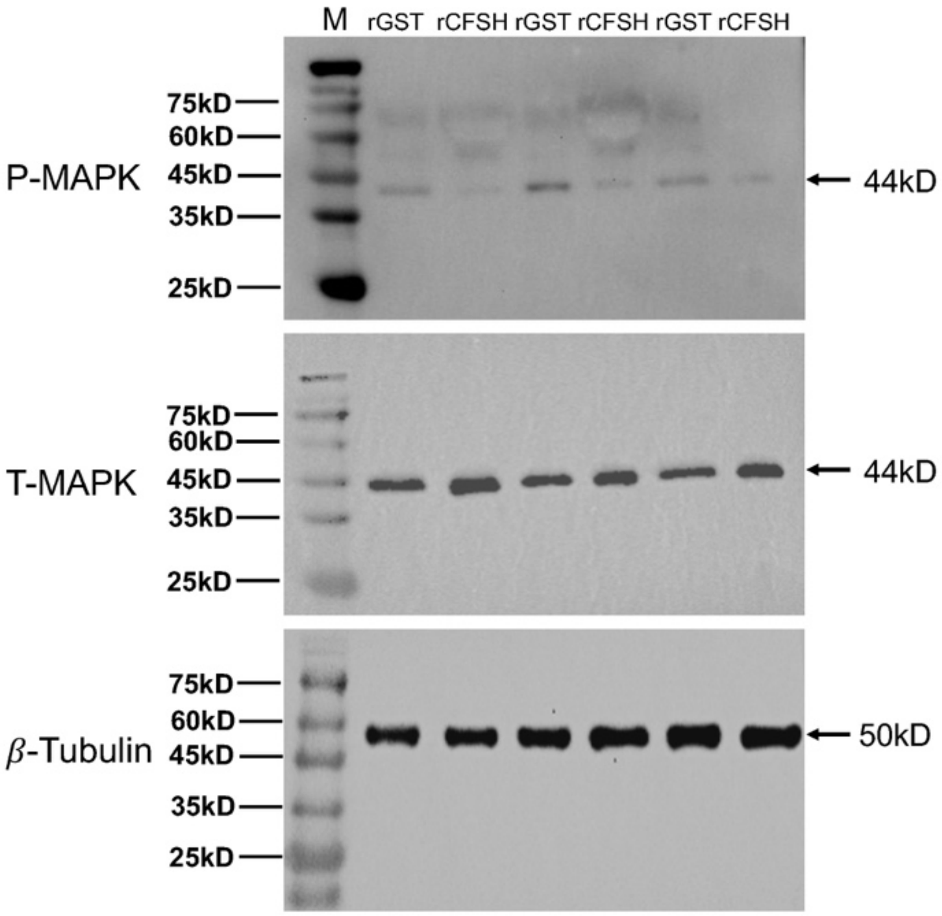

Supplemental Figure S3. Phosphorylated-MAPK was detected in testis after long-term in vivo injection of rPtCFSH. Total-MAPK and β-tubulin were used as internal reference. Arrows indicate the size of protein molecules.

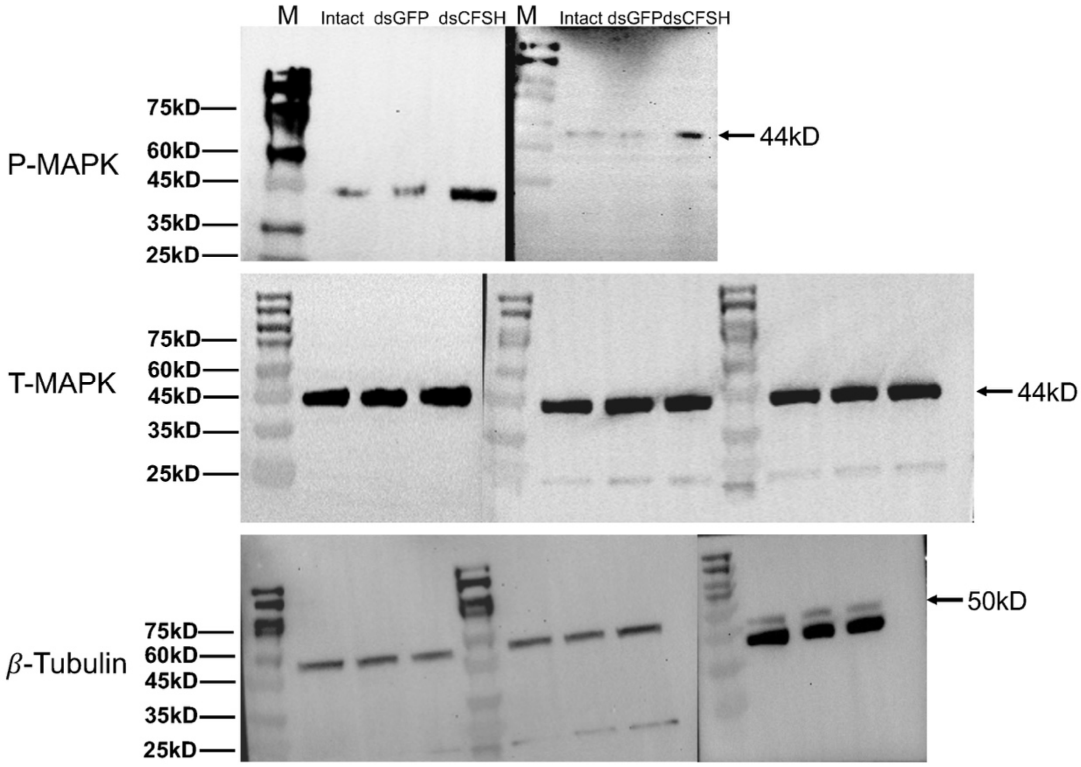

Supplemental Figure S4. Phosphorylated-MAPK was detected in testis after long-term in vivo injection of dsPtCFSH, dsGFP. Total-MAPK and β-tubulin were used as internal reference. Arrows indicate the size of protein molecules.

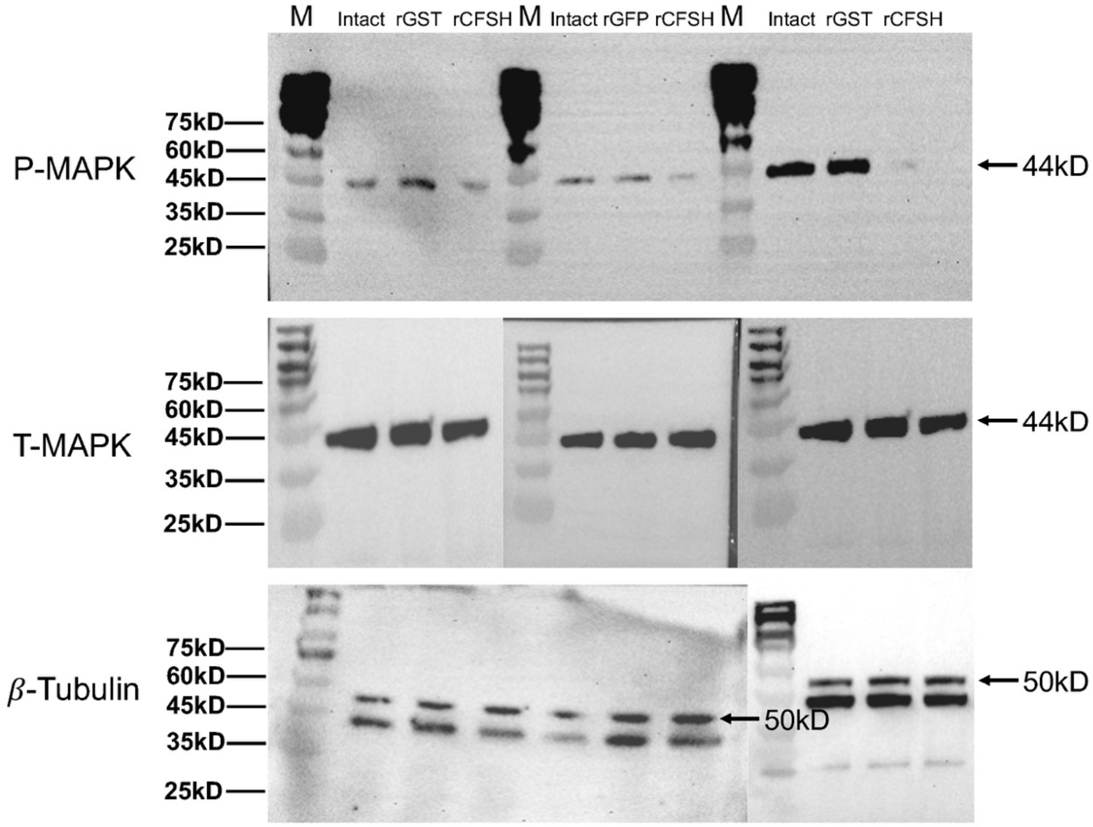

Supplemental Figure S5. Phosphorylated-MAPK was detected in stestis after incubation of teitis in vitro and addition of rPtCFSH and rGST, respectively. Total-MAPK and β-tubulin were used as internal reference. Arrows indicate the size of protein molecules.

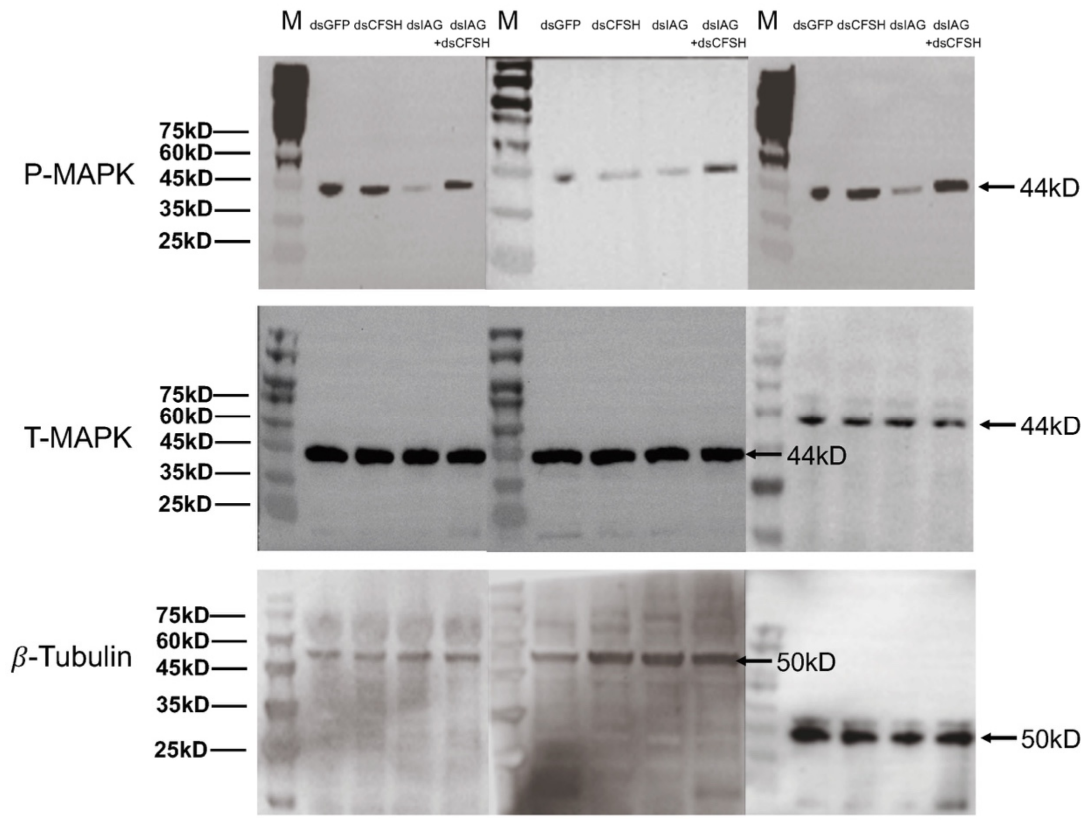

Supplemental Figure S6. Phosphorylated-MAPK was detected in testis after short-term in vivo injection of dsCFSH, dsIAG, dsIAG followed by dsCFSH, and dsGFP, respectively. Total-MAPK and β-tubulin were used as internal reference. Arrows indicate the size of protein molecules.

Supplemental Table S1

Table S1. Primers used in this study

Name	Sequence（5′-3′）	PCR objective
PtCFSH-F1	CGTCAAATACAAGGACCGCTC	3' RACE
PtCFSH-F2	CAATGTTCTCAAGATGGGTCC	3' RACE
PtCFSH -R1	CCTCGCAGTCCAGAACCAA	5' RACE
PtCFSH -R2	GTGATAGAGGGCGTGGG	5' RACE
PtCFSH-F	TCCTATTGGAGTGTTCATTCGC	cDNA clone
PtCFSH-R	GTGGTGGTACAGTTGGTGGG	cDNA clone
PtCFSH-RT-F	ACCGCCTACCAGTATGGATTAG	RT-PCR
PtCFSH-RT-R	GCATCAGCAACAACAGCAGTA	RT-PCR
5'outer	CTAATACGACTCACTATAGGGC	5' RACE
5'inner	AAGCAGTGGTATCAACGCAGAGT	5' RACE
3'inner	TCCACTAGTGATTTCACTATAGG	3' RACE

3'outer	CTAATACGACTCACTATAGGGC	3' RACE
AP	TACCGTCGTTCCACTAGTGATTTCACTATAGG(T)17	3' RACE
dsGFP-F	TAATACGACTCACTATAGGGCGACGTAAACGGCCACAAGT	RNAi
dsGFP-R	TAATACGACTCACTATAGGGCTTGTACAGCTCGTCCATGC	RNAi
dsPtCFSH-F	TAATACGACTCACTATAGGGAGATCCTATTGGAGTGTTCATTCG	RNAi
dsPtCFSH-R	TAATACGACTCACTATAGGGAGATACAGTTGGTGGGTGAGTCG	RNAi
dsPtIAG-F	TAATACGACTCACTATAGGGAAACGAAGACCCAATGCTACC	RNAi
dsPtIAG-R	TAATACGACTCACTATAGGGTTACTGCCTATTTCGGGAAGC	RNAi
PtCFSH-QF	GTATTTCATCTTAGGATGCCAA	qRT-PCR
PtCFSH-QR	TAAACTCTGCCCTTCATTTTCT	qRT-PCR
β-actin-QF	CGAAACCTTCAACACTCCCG	qRT-PCR
β-actin-QR	GATAGCGTGAGGAAGGGCATA	qRT-PCR
PtIGFBP-rp-QF	TTACCACTATTGACGGCACCT	qRT-PCR
PtIGFBP-rp-QR	TCATTATC TGTACCCATCCTGTT	qRT-PCR
PtIAG-QF	TCTTATTAGCGACTTCTCCG	qRT-PCR
PtIAG-QR	CCTCTGTCCCTCGTTTATGT	qRT-PCR
PtIR1-QF	CTGATGCGTTTGTCGTATTT	qRT-PCR
PtIR1-QR	GAAGCGTGGTGCCTATTT	qRT-PCR
PtIR2-QF	ACCAGCTAGTGGGAACCG	qRT-PCR
PtIR2-QR	GGGAGGGACTCTTTGACG	qRT-PCR
PtAkt-QF	CTCAACCAGGAACGCTTCTTC	qRT-PCR
PtAkt-QR	TGTGTCCATCAGCATCCAGTAA	qRT-PCR
PtmTOR-QF	TCTCCTGGCTGTTGCTGTC	qRT-PCR
PtmTOR-QR	GCTTCTTGCTTGGTGTATCCTT	qRT-PCR
PtAkt-QF	CTCAACCAGGAACGCTTCTTC	qRT-PCR
PtAkt-QR	TGTGTCCATCAGCATCCAGTAA	qRT-PCR
Ptcdc2-QF	CCGTCAAGCAGATGGACAGTG	qRT-PCR
Ptcdc2-QR	CCAGGTCGTCAAAGTAAGGGTG	qRT-PCR
PtCyclinB-QF	ATGTGCCACTACAAGGCGTCT	qRT-PCR
PtCyclinB-QR	ATCAGCGTGTCATTCCAATCC	qRT-PCR
PtFoxo-QF	CGGAGGTGAAGCACATCAAC	qRT-PCR
PtFoxo-QR	TCATTGGTGGAGGCAGAGTG	qRT-PCR
PtKifc1-QF	TCCAATCGCCATCTACCTCAG	qRT-PCR
PtKifc1-QR	CGTCTTCAGCATCTCCAGAATG	qRT-PCR
PtVasa-QF	GCTTGCCATCCAGATATTCCAT	qRT-PCR
PtVasa-QR	TGCTCCTTCATACGCCTCAA	qRT-PCR
PtCFSH-GST-SmaI-F	ggatccccaggaattcccgggATGAAGCAGAACGGAGCTTCTC	In-Fusion clone
PtCFSH-GST-XhoI-R	gtcacgatgcggccgctcgagTCATTTATTCTCGCTTAAGTCAATATAGC	In-Fusion clone

Supplemental Table S2

Table S2. List of species used in multiple sequence comparison phylogenetic analysis

Species	Protein name	GenBank Accession number
Portunus trituberculatus	CFSH	ON929327
Scylla paramamosain	CFSH	MN938502.1
Callinectes sapidus	CFSH	GU016328.1
Carcinus maenas	CFSH-1	AEI72264.1
Marsupenaeus japonicus	CFSH	BBA53799.1
